# Effects of Flapless Laser Corticotomy in Upper and Lower Canine Retraction: A Split-mouth, Randomized Controlled Trial

**DOI:** 10.7759/cureus.37191

**Published:** 2023-04-06

**Authors:** Abubakr R Bakr, Mohamed A Nadim, Youssef W Sedky, Abbadi A El Kady

**Affiliations:** 1 Department of Orthodontics, Faculty of Dentistry, Suez Canal University, Ismailia, EGY; 2 Department of Orthodontics, Faculty of Oral and Dental Medicine, Misr International University, Cairo, EGY

**Keywords:** accelerated orthodontics, cone-beam computed tomography (cbct), tads, temporary anchorage device, root resorption, canine retraction, 3d digital models, flapless laser corticotomy, bimaxillary protrusion

## Abstract

Aim

One of the major difficulties in orthodontic treatment is the lengthy course of therapy, particularly in situations involving extractions. Hence, various methods for accelerating tooth movement rate had been devised. Flapless corticotomy is one of those methods. This study aimed to evaluate the effects of flapless laser corticotomy (FLC) compared to the conventional retraction (CR) method on the rate of canine retraction.

Methods

A split-mouth, randomized controlled trial included 56 canines from 14 patients (12 females and two males) with a mean age of 20.4 ± 2.5 years, who were complaining of bimaxillary protrusion requiring extraction of four premolars. All canines were randomly assigned to four groups (maxillary FLC, maxillary control CR, mandibular FLC, and mandibular control CR). Randomization was performed by creating two equal, random computer-generated lists with a 1:1 allocation ratio-one list for the right side and one for the left. The allocation concealment was achieved using opaque sealed envelopes until the time of intervention. FLC was applied on the experimental sides before canine retraction by drilling six holes penetrating 3 mm into the bone on the mesial and distal sides of the canines. Subsequently, all canines were retracted employing closed coil springs to deliver a force of 150 g using indirect anchorage from temporary anchorage devices (TADs). All canines were assessed at T0 (before retraction), T1 (one month after retraction), T2 (two months), and T3 (three months) using three-dimensional (3D) digital models. Additionally, canine rotation, molar anchorage loss assessed using 3D digital models, root resorption assessed using cone beam computed tomography (CBCT), probing depth, plaque, gingival indices, and pulp vitality were all evaluated as secondary outcomes. It was possible to blind only the outcome analysis expert (single-blinded).

Results

The measurements of canine retraction during the follow-up period from T0 to T3 were 2.46 ± 0.80 mm and 2.55 ± 0.79 mm in maxillary FLC and control groups, respectively, and 2.44 ± 0.96 mm and 2.31 ± 0.95 mm in mandibular FLC and control groups, respectively. The results demonstrated a statistically non-significant difference in the distance of canine retraction between the FLC and control groups at all time points. Moreover, no differences were observed between groups in canine rotation, molar anchorage loss, root resorption, probing depth, plaque, gingival indices, and pulp vitality (p > 0.05).

Conclusion

In the FLC procedure performed in this study, the rate of upper and lower canine retraction could not be accelerated and no significant differences were observed between FLC and control groups in canine rotation, molar anchorage loss, root resorption, periodontal condition, and pulp vitality.

## Introduction

The prolonged duration of active treatment is one of the most frequent problems in orthodontic therapy, particularly in extraction cases [1]. Since almost every orthodontic patient asks about the possibility of reducing treatment time, the need for accelerating orthodontic teeth movement has become highly warranted. The following approaches to accelerate tooth movement have been attempted: (1) Pharmacological and chemical agents such as vitamin D3, corticosteroids, and prostaglandins administered locally and systemically [2,3], (2) Physical agents concomitant with orthodontic force to augment mechanical force, for example, local heat application, electric current, and static magnetic field [4] and low-level laser therapy (LLLT) [5], and (3) Surgical approaches to accelerate tooth movement using surgical burs, vertical grooves, and/or perforations in the cortical plate (alveolar corticotomy) [6-8] in conjunction with conventional retraction methods or through dentoalveolar distraction osteogenesis (DADO) [9].

Corticotomy or decortication refers to the intentional cutting of the bony cortical layer. This procedure has been claimed to shorten treatment duration because the resistance from the cortical bone to orthodontic tooth movement is eliminated [10-13]. This decreased resistance to tooth movement has been explained by the provocation of the underlying regional acceleratory phenomenon (RAP) that occurs after a bony surgical insult. RAP involves the recruitment of osteoblasts and osteoclasts to the injury site for healing wounds, which, in turn, leads to transient localized demineralization and remineralization in the dentoalveolar bony housing [14]. Corticotomy has been claimed to be an effective means of tooth movement acceleration [6,11]. However, it is considered to be an invasive procedure as the patient is subjected to flap reflection, bone drilling, cutting by burs, and suturing, and these procedures are accompanied by complications such as contamination, pain, and swelling [15-18].

To overcome these complications, many researchers have tried developing other minimally invasive alternatives by performing corticotomy without raising the flap (flapless corticotomy) [19,20], like corticision [21-23], piezocision [24-26], micro-osteoperforation (MOPs) [27,28], and flapless laser corticotomy (FLC) [29-33]. 

Few studies have investigated the FLC technique to elaborate on its effectiveness. The first animal study that employed high-intensity erbium, chromium-doped yttrium, scandium, gallium, and garnet (Er-Cr: YSGG) laser to perform FLC on rabbits showed that laser-facilitated flapless corticotomy was a helpful procedure to expedite the treatment time by avoiding the necessity of a more invasive flap surgery [29]. Further studies on humans revealed similar results but with different degrees of effectiveness and without recommending a standard protocol for this procedure [30-33].

A recent systematic review conducted by Shaadouh et al. furnished low to moderate evidence supporting the efficacy of FLC in the acceleration of orthodontic tooth movement, at least in the first two months [34]. Moreover, they suggested the need for more properly conducted, high-quality randomized controlled trials (RCTs) to confirm these results. 

Specific objectives and hypotheses

This study aimed to evaluate the following: (i) Primary outcome: To evaluate the effect of FLC on the rate of upper and lower canine retraction; (ii) Secondary outcomes: To evaluate the difference in terms of the following (possible side effects) between FLC, control maxillary, and mandibular canine groups: canine rotation, molar anchorage loss, root resorption, periodontal condition, and pulp vitality conditions.

## Materials and methods

Trial design

The study was designed as a split-mouth, RCT with an allocation ratio of 1:1, following the Consolidated Standards Of Reporting Trials (CONSORT) statement guidelines (evidence-based, minimum set of recommendations for reporting randomized trials; www.consort-statement.org). A split-mouth design was adopted to ensure more accurate results by eliminating inter-subject variability and using a small-sized sample as the sample set-up for each patient to act as their control. There were no changes in the methods after trial initiation.

Participants, eligibility criteria, and settings

The study methods were approved by the Research Ethics Committee, Faculty of Dentistry, Suez Canal University, Ismailia, Egypt (approval number: 46/2017). Moreover, the study was registered at ClinicalTrials.gov with an identifier number NCT04631419. The participants were selected from the outpatient clinic of the orthodontic department of the Faculty of Dentistry, Suez Canal University, Ismailia, Egypt. The sample included 14 patients (12 females and two males), with a mean age of 20.4 ± 2.5 years. This single group of 56 canines (28 maxillary and 28 mandibular) was divided into four tails as follows: (i) Maxillary FLC group: FLC was randomly applied to one side of all upper arches (experimental side) before canine retraction, (ii) Maxillary control group: Canine retraction was performed on the other side without FLC, (iii) Mandibular FLC group: FLC was randomly applied to one side of all lower arches (experimental side) before canine retraction, and (iv) Mandibular control group: Canine retraction was performed on the other side without FLC. 

The inclusion criteria applied were: (i) Age ≥18 years for both male and female subjects, (ii) Class I with bimaxillary dentoalveolar protrusion with minimum or no crowding, requiring four first premolars extraction and maximum anchorage, (iii) No history of previous orthodontic treatment, (iv) Healthy people with no history of any craniofacial deformities, chronic systemic illness, or syndromes, (v) Vital teeth with no signs of root resorption, and (vi) Adequate oral hygiene; probing depth values not exceeding 3 mm, with the attached gingiva having an adequate thickness (1-2 mm) across the entire dentition. Exclusion criteria were: (i) Systemic diseases that might affect bone formation or density, such as osteoporosis, (ii) The presence of any systemic or local surgical contraindication, (iii) Extracted or missing permanent teeth, except for third molars, and (iv) Facial asymmetry.

After recruitment, patients were examined and selected according to the inclusion and exclusion criteria, following which they were explained the intervention and associated risks. Informed signed consent forms were obtained from the patients before enrollment in the study. The subjects of this study were recruited between June 2018 and August 2019, and the final data collection took place in December 2020.

Sample size calculation

The sample size was calculated using the G power software (Universität Düsseldorf, Germany), which was used to calculate the sample size on the basis of a type 1 error of 5%, with an alpha value of 0.05 and a power (1-β) of 0.80 with an estimated effect size d of 1 calculated based on a previous study [30]. This revealed the need for a sample of 14 subjects per group (i.e., a total sample of 56 canines). 

Randomization

To select the side of the experimental intervention, all patients were assigned numbers and then two equal, random computer-generated lists with a 1:1 allocation ratio were generated by the Random.org website (https://www.random.org/), with the first list for the right side and the second for the left side. Allocation concealment was achieved using opaque sealed envelopes until the time of intervention to avoid selection bias. The patients were asked to detect their number on the right side or left side lists on the day of the intervention. 

Blinding

Blinding of the operators and patients was not possible. The results of the experiment were analyzed blindly by an external statistics expert (single-blinded). 

Interventions

Orthodontic Treatment Steps

**Figure 1:**
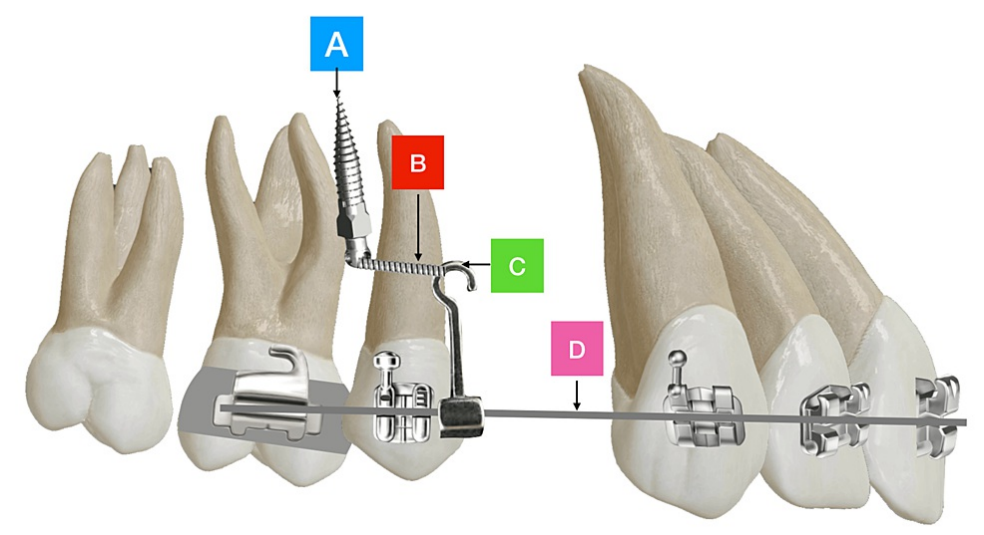
Illustration shows (A) Mini-screw between second premolar and first molar, (B) Stainless steel ligature wire, (C) Crimpable hooks, (D) 0.017 x 0.025 stainless steel working arch wire.

All patients underwent orthodontic treatment therapy by the same operator using a fixed upper and lower pre-adjusted orthodontic appliance (TRAX Plus; Ortho Pro Dent LLC, Orlando, Florida, USA) with slot size (0.022") MBT prescription. Alignment proceeded until 0.019 x 0.025" Nickel-titanium (NiTi) archwire, following which the patients were referred to the extraction of all first premolars. After three months of extraction, the working archwire of 0.017 x 0.025" stainless steel (SS) was installed and long crimpable hooks (PARVUS^TM^; MATT Orthodontics, LLC, Chicago, Illinois, United States) were crimped to the archwire mesial to second premolar brackets. Self-drilling temporary anchorage devices (TADs) 1.6 × 8 mm (MATT Orthodontics, LLC) were placed buccally between the second premolars and the first molars bilaterally in the upper and lower arches. Thereafter, they were attached to the crimpable hooks by a 0.010 SS ligature to provide maximum indirect anchorage. Flowable composite was added to the top of the hook to reduce the discomfort (Figure 1).

FLC Technique

The experimental sides to be subjected to FLC were determined according to the previously prepared random lists, which were concealed until the time of intervention. A guide made of 0.019 x 0.025" SS wire that was marked at intervals of each 2 mm was used to determine the points of laser application. Before the laser application, the surgical area was disinfected with Betadine solution, and local anesthesia was administered with articaine hydrochloride 4% 1:100.000 (Artinibsa, Laboratorios Inibsa, S.A., Barcelona, Spain). The FLC was performed by a laser expert operator using an Er, Cr: YSGG Laser device (Waterlase iPlus®; BIOLASE, Inc., California, United States), with a wavelength of 2780 nm and a sapphire tip MZ8 (800 microns diameter and 6 mm length).

To cut a series of circular holes along the planned positions inside the bone with the help of the custom-made guide, the first hole was made 6 mm above the bracket slot level. Thereafter, three mesial holes that were 2 mm apart and three distal holes were created parallel to the long axis of the experimental sides of the canine’s roots. For soft tissue cutting, the tip was brought in contact with the tissues at 90 degrees angulation. Laser parameters were set as the average power of 2.5 W, pulse repetition rate was 40 Hz, energy per pulse was 62.5 MJ, and pulse duration was set for H-mode (60 microseconds), making a peak power of 1041 W, with 20 air:40 water concentration. When approaching the bone, the tip was held in non-contact mode, the average power was raised to 4.5 W at a pulse repetition rate of 40 Hz, energy per pulse was 112.5 MJ, and the pulse duration was set for H-mode (60 microseconds), making a peak power of 1875 W and, with 20 air:40 water concentration to improve laser absorption.

**Figure 2:**
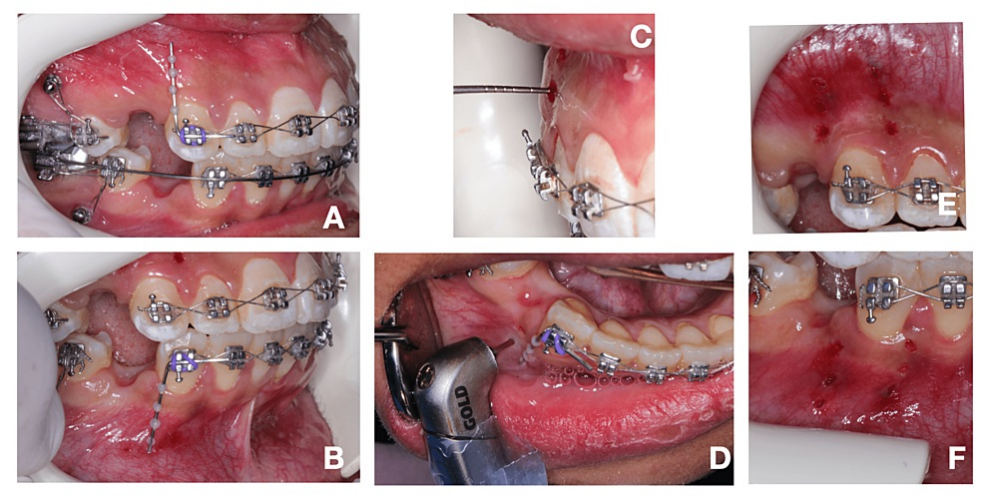
(A) Maxillary and (B) mandibular custom-made guides; (C) Periodontal probe to measure the depth of holes; (D) Flapless laser corticotomy by laser hand piece; (E) Completed flapless laser corticotomy mesial and distal holes in upper and (F) lower canines before retraction.

The depth of laser cutting was measured to reach 3 mm during the surgery using a periodontal probe read to reach 3 mm depth into the medullary bone to enhance bleeding, as illustrated in Figure 2 (A-D). After the intervention, patients were provided instructions to use chlorohexidine 0.2% mouthwash twice a day for one week to control the infection and take acetaminophen 500 mg tablet when needed to control postoperative pain.

Canine Retraction

**Figure 3:**
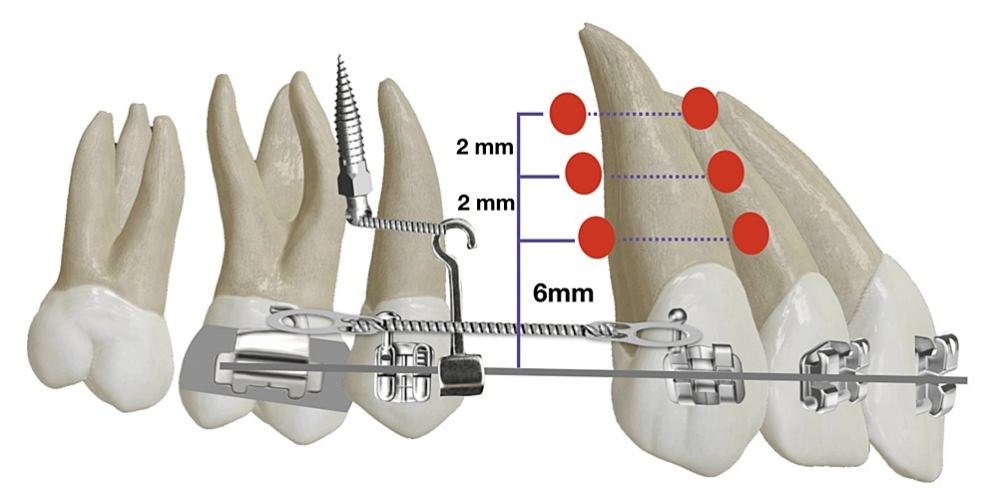
Orthodontic appliance set-up and laser application sites

**Figure 4:**
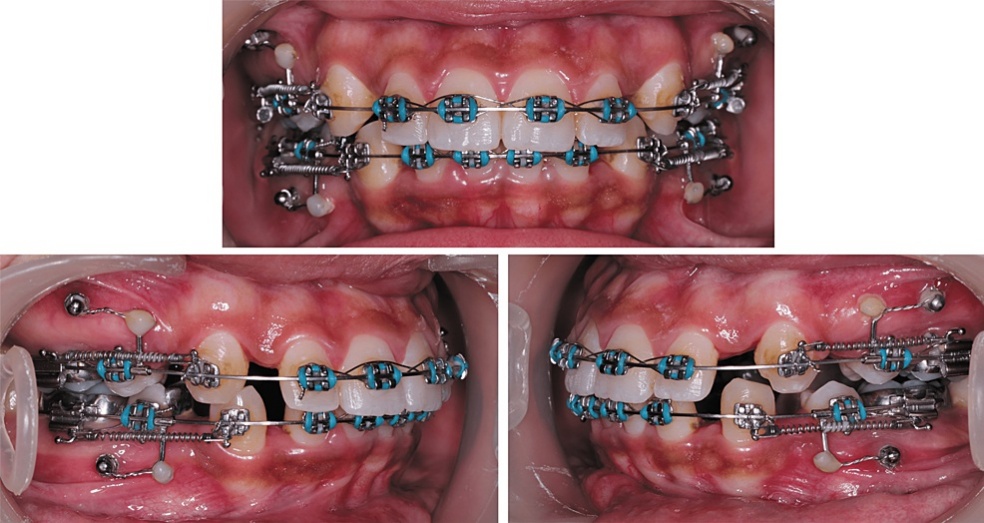
Canine retraction procedure.

After the FLC procedure, all canines were ligated with SS ligature, following which a closed coil spring (9mm; MATT Orthodontics, LLC) was attached between the canine bracket hook and the first molar band hook as demonstrated in (Figures 3, 4). The coil spring was activated to deliver 150 gm of force, which was confirmed with force gauge (DTC Orthodontics, Hangzhou, China). The patients were followed up every two weeks to ensure consistent force levels throughout the study duration. 

Outcomes assessment

Primary Outcomes

As the primary aim was to assess the rate of canine retraction, this was achieved using indirect measurements on digital models. Upper and lower alginate impressions and the wax bite in centric occlusion were taken at four time points: just before canine retraction (T0), one month after the procedure (T1), two months after the procedure (T2), and three months after the procedure (T3). These alginate impressions were immediately poured to fabricate study models, following which they were scanned using AutoScan-DS-EX (SHINING 3D Tech Co., Ltd., Zhejiang, China) desktop scanner to export all data in the standard triangle language (STL) format and create digital models. All digital models were manipulated using the software OrthoAnalyzer 2020 (3Shape, Copenhagen, Denmark).

**Figure 5:**
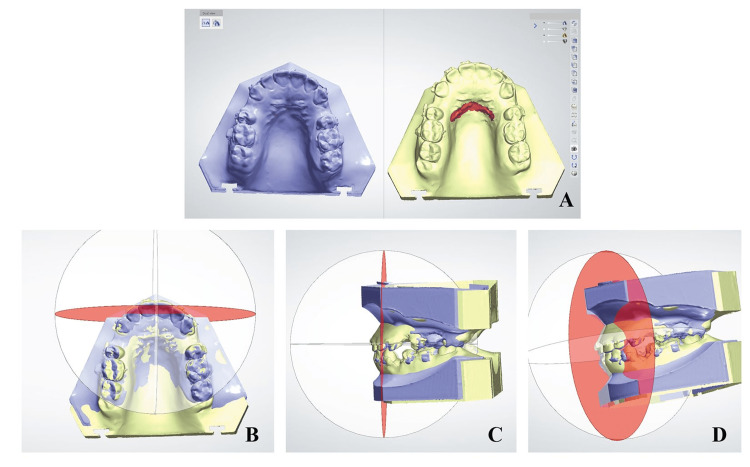
(A) Different time points, three-dimensional digital models, and superimposition using third rugae as a stable landmark; (B) Constructed antero-posterior plane (red) from occlusal view that was used as a common reference plane for upper and lower superimposed models; (C) Lateral view; (D) three-dimensional view.

To obtain a standardized measurement method, a reference plane was required to be made. Therefore, the anterior-posterior (AP) plane was selected for this purpose. The upper arch of the T0 digital model was used to construct the occlusal, sagittal, and AP planes. Thereafter, on the occlusal view of the model, the AP plane was reoriented such that it was perpendicular to the sagittal plane and passed through a constructed point representing the distal end of the incisive papilla. The subsequent T1, T2, and T3 models were superimposed on the T0 model using the third rugae area. The superimposition was performed with the option of keeping the occlusion selected, such that the software superimposed the upper and lower casts of all time points on the same landmarks (Figure 5 A). Furthermore, the first constructed AP plane could then be used as a standard reference plane for all upper and lower models (Figure 5 B-D).

**Figure 6:**
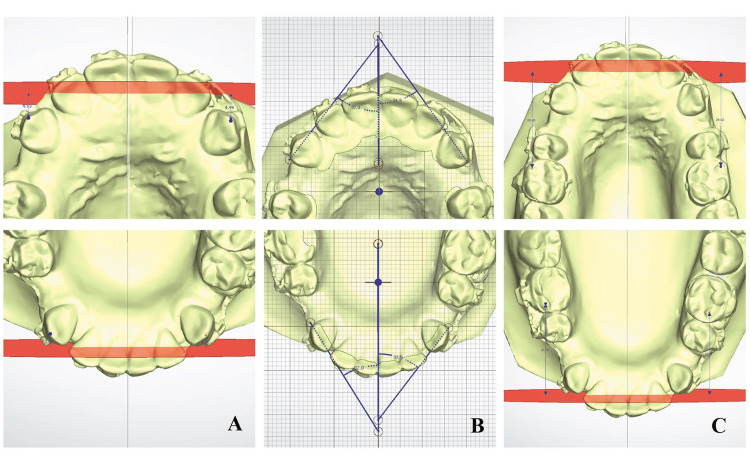
(A) Upper and lower canine retraction measurement from the cusp tip to the antero-posterior plane; (B) Canine rotation measurements from the angle between the midsagittal plane and the line connecting the mesial and distal contact points of canine; (C) Molar anchorage loss from mesiobuccal cusp tip to the antero-posterior plane.

The distance between the AP plane and the upper and lower canine tips on both the experimental and control sides were recorded at T0, T1, T2, and T3 to calculate the monthly rate of canine retraction (Figure 6 A). 

Secondary Outcomes

Canine rotation was measured as the angle between a line connecting the distal and mesial contact points of canines and the sagittal plane in all canines at all time points (Figure 6 B). Molar anchorage loss was measured on superimposed models as the distance between the AP plane and the mesiobuccal cusp tip of all molars at all time points (Figure 6 C).

Canine root resorption

Cone beam computed tomography (CBCT) scans were obtained for all patients before the start of treatment and immediately after finishing the canine retraction with SCANORA® 3Dx (SOREDEX, Tuusula, Finland). The CBCT device was set to 90 kVp/4-12.5mA and the exposure time was 2.4-6 seconds. Dimensions of the volume were 330 x 330 x 480, with a resolution of 0.5 x0.5 x 0.5 mm. All data were imported in the digital imaging and communications in medicine (DICOM) format and then manipulated by Dolphin Imaging Software Version 11.95 (Dolphin Imaging & Management Solutions, Chatsworth, California, United States).

**Figure 7:**
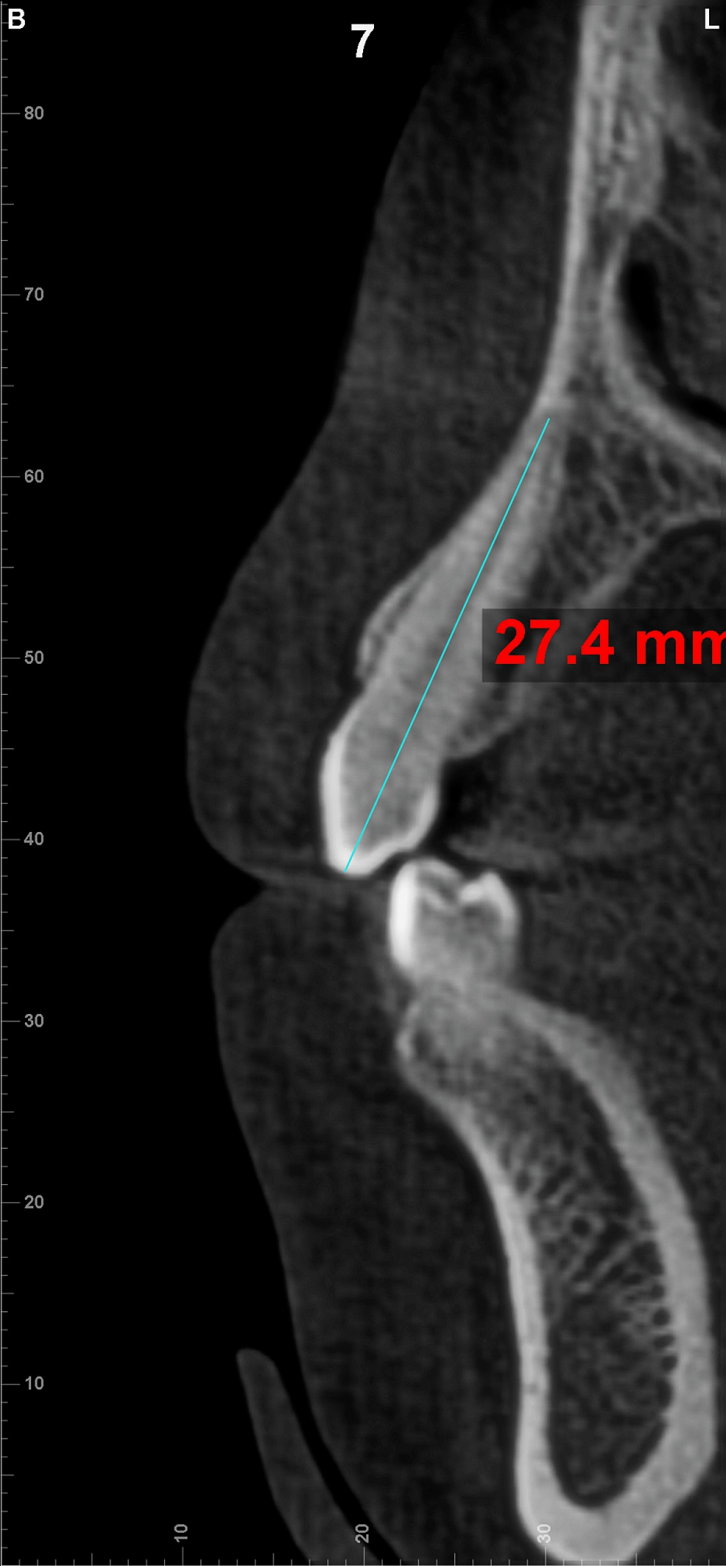
Canine root length measurement from CBCT. CBCT: cone beam computed tomography

The pretreatment panoramic and lateral cephalometric views were extracted from CBCT, and the same software was utilized to measure canine root length before and after treatment. To measure canine root resorption, the CBCT volume was reoriented to make the sagittal plane pass through the long axis of the canine for inspection. Thereafter, the same procedure was repeated with all upper and lower canines in the initial and post-retraction CBCT scans to record the maximum length of the canines, which was measured from the cusp tip to the apex of the root in the cross-section view (Figure 7).

Periodontal assessment

Periodontal assessment of canines before and after retraction was evaluated through gingival index, plaque index, and probing depth according to the method of Löe [35] and Armitage [36]. The assessment was performed using a periodontal probe to assess four different gingival areas around the canine and provide average scores. Probing depth was measured using a graduated Williams periodontal probe from the free gingival margin to the depth of the periodontal sulcus. Plaque index was obtained through inspection and by running a probe around the gingival part of the canine crown to give a score from 0 to 3, where 0 indicated no plaque accumulation in the gingival area and 3 indicated abundance of soft matter within the gingival margins. The gingival index was assessed through a periodontal probe to detect bleeding upon probing and a score was given from 0 to 3, where 0 indicated normal gingiva with no bleeding and 3 indicated severe inflammation with marked redness and edema with a tendency to spontaneous bleeding.

Vitality test

Vitality test was performed for all canines before and immediately after canine retraction using a cold thermal test by applying the water-based spray Endo-Ice (Maquira Dental Group, Paraná, Brazil) at a temperature of −50°C by first spraying on a cotton pellet and then applying to the tooth surface before and immediately after canine retraction to record pulp response, which indicates vitality.

Statistical analysis

Data were analyzed using IBM SPSS Statistics for Windows, Version 20.0 (Released 2011; IBM Corp., Armonk, New York, United States). ANOVA test with repeated measures for normally distributed quantitative variables was performed to compare more than two periods or stages and post hoc test (Bonferroni adjusted) for pairwise comparisons was conducted for drawing a comparison between the different time points within each group according to the change in canine retraction distance from T0 (mm). Student t-test was used to compare FLC and control groups in terms of canine retraction change from T0, monthly rate, canine rotation, molar anchorage loss, root resorption, and probing depth at each time point. Additionally, paired t-test was used to analyze root resorption and probing depth before and after canine retraction within each group. The plaque index and gingival indices were analyzed using the Wilcoxon signed-rank test, which was employed for comparing different time points in each group. The Shapiro-Wilk test was utilized to verify the normality of distribution. Quantitative data were described using mean, standard deviation, range (minimum and maximum), and median. The significance of the obtained results was judged at the 5% level.

Measurements pertaining to canine retraction, canine rotation, anchorage loss, and root resorption were measured again by the same operator one month after the first measurements to perform the intraclass correlation coefficient to test intra-examiner reliability. The results highlighted a strong agreement between reading 1 and reading 2 (intraclass correlation coefficient (ICC) ranged from 0.985-0.999).

## Results

Participant flow

**Figure 8:**
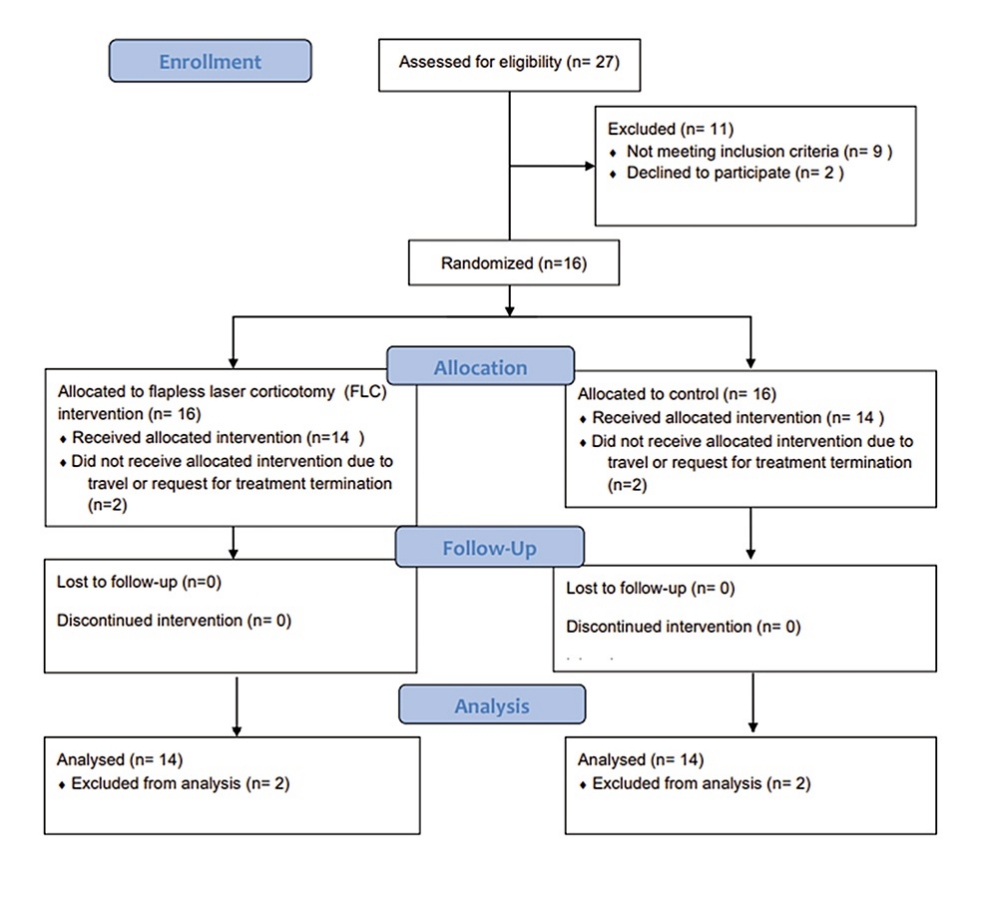
CONSORT patient flow diagram CONSORT: Consolidated Standards Of Reporting Trials

Patient flow is shown in Figure 8. Sixteen patients were initially enrolled in the study; however, two were excluded before starting the extraction and FLC intervention, as one patient requested to discontinue the treatment and the other was unable to follow up as he traveled to another country. The final group of enrolled patients comprised 14 patients (12 females and two males) who successfully finished the intervention and follow-up period. A follow-up period of three months was selected, as most acceleration effects would occur during this period before the rate of orthodontic movement declined to the pre-surgical level.

Primary outcome

A total of 56 canines (14 canines per group) were analyzed. Two patients reported signs of failure in one of the TADs in the form of slight mobility, inflammation, and pain within days from insertion or follow-up visits. These patients were recalled, and the TAD was reinserted between the first and second molars on the same side. Retraction was continued immediately after adjusting force by adding a ligature wire to the coil spring and measuring the applied force. Baseline data are presented in Table 1.

**Table 1:** Baseline characteristics of the sample SD: Standard deviation; n: number; SNA: Sella-Nasion-A point angle; SNB: Sella-Nasion B point angle; ANB: A point-B point angle; MMP: maxillary mandibular plane angle; U1: upper incisors; SN: Sella-Nasion plane; L1: lower incisor; MnP: mandibular plane

	n (%)
Male	2 (14.3 %)
Female	12 (85.7 %)
	Mean ± SD
Age	20.45 ± 2.5
SNA	84.67 ± 3.94
SNB	78.53 ± 3.18 ˚
ANB	3.77 ± 3.27 ˚
MMP	35.65 ± 4.5 ˚
U1 to SN	112.1 ± 4.72 ˚
L1 to MnP	102.5 ± 4.84 ˚
Overjet	3.91 ± 0.89 mm
Overbite	1.25 ± 1.32 mm

The results revealed that the amount of canine retraction during the follow-up period from T0 to T3 at (p < 0.001) was 2.46 ± 0.80 mm and 2.55 ± 0.79 mm in the maxillary FLC and control groups, respectively and 2.44 ± 0.96 mm and 2.31 ± 0.95 mm in the mandibular FLC and control groups, respectively. However, a statistically non-significant difference was observed between FLC and control sides in canine retraction distance at all time points from T0 to T1 (upper p = 0.18/lower p = 0.71), T2 (upper p = 0.36/lower p = 0.78), and T3 (upper p = 0.79/lower p = 0.74) as demonstrated in Table 2. Moreover, the results highlighted non-significant differences in the monthly rate of canine retraction between FLC and control sides in the first month (upper p = 0.19/lower p = 0.71), the second month (upper p = 0.66/lower p = 0.91), and third month (upper p = 0.16/lower p = 0.88) (Table 3).

**Table 2:** Comparison between different groups in terms of the change in canine retraction distance from T0 at one, two, and three months. Student t-test, T1: baseline to the first month; T2: baseline to the second month; T3: baseline to the third month SD: standard deviation; CI: confidence interval; LL: lower limit; UL: upper limit; CR: conventional retraction; FLC: flapless laser corticotomy

		CR Control Mean (mm)± SD	FLC Mean (mm) ± SD	p-value	Mean Difference	95% CI	
						LL	UL
Upper canines	T1	0.51 ± 0.22	0.62 ± 0.15	0.18	0.11	−0.05	0.27
	T2	1.42 ± 0.43	1.63 ± 0.65	0.36	0.21	−0.26	0.67
	T3	2.55 ± 0.79	2.46 ± 0.80	0.79	−0.09	−0.76	0.59
Lower canines	T1	0.54 ± 0.34	0.59 ± 0.27	0.71	0.05	−0.21	0.31
	T2	1.39 ± 0.57	1.47 ± 0.74	0.78	0.08	−0.48	0.59
	T3	2.31 ± 0.95	2.44 ± 0.96	0.74	0.13	−0.68	0.94

**Table 3:** Comparison between different groups in terms of the monthly rate of canine retraction (mm) Student t-test; T0-T1: baseline to the first month; T1-T2:  first month to the second month; T2-T3: second month to the third month SD: standard deviation; CR: conventional retraction; FLC: flapless laser corticotomy

		CR Control Mean (mm)± SD	FLC Mean (mm) ± SD	p-value	Mean Diff mm	95% CI	
						LL	UL
Upper canines	First month T0-T1	0.51 ± 0.22	0.62 ± 0.15	0.19	0.11	-0.06	0.27
	Second month T1-T2	0.91 ± 0.41	1.01 ± 0.68	0.66	0.1	-0.06	0.52
	Third month T2-T3	1.13 ± 0.60	0.84 ± 0.36	0.16	-0.29	-0.6	0.01
Lower canines	First month T0-T1	0.54 ± 0.34	0.59 ± 0.27	0.71	0.05	-0.18	0.27
	Second month T1-T2	0.85 ± 0.61	0.88 ± 0.65	0.91	0.03	-0.25	0.31
	Third month T2-T3	0.92 ± 0.77	0.97 ± 0.94	0.88	0.05	-0.27	0.56

Secondary outcomes

The results revealed a canine rotation of approximately 10.49 ± 4.66 degrees and 11.82 ± 3.53 degrees in the maxillary FLC and control groups, respectively and 11.55 ± 3.31 degrees and 9.90 ± 1.77 degrees in the mandibular FLC and control groups, respectively. Regarding molar anchorage loss, upper molars showed forward movement of 1.11 ± 0.26 mm in the control group and 0.81 ± 0.34 mm in the FLC group, and mandibular molars showed 1.24 ± 0.60 mm in the control group and 0.98 ± 0.41in the FLC group. The analysis indicated statistically non-significant differences between the control and FLC groups in upper and lower canine rotation and molar anchorage loss at different time points (p > 0.05), except for the third-month measurements of upper arches where the control group showed statistically significant more anchorage loss than the FLC group by 0.3 mm (p = 0.023) (Table 4).

**Table 4:** Comparison between different groups in terms of canine rotation and molar anchorage loss from T0 at one, two, and three months Student t-test; T0: baseline; T1: baseline to first month; T2: baseline to second month; T3: baseline to third month SD: standard deviation; CI: confidence interval; LL: lower limit; UL: upper limit; CR: conventional retraction; FLC: flapless laser corticotomy *Statistically significant at p ≤ 0.05

Canine rotation from T0									Molar anchorage loss from T0					
			CR Control Mean (degree)± SD	FLC Mean (degree) ± SD	p-value	Mean Diff degrees	95% CI		CR Control Mean (mm) ± SD	FLC Mean (mm) ± SD	p-value	Mean Diff mm	95% CI	
							LL	UL					LL	UL
Upper canines	T1		3.49 ± 2.91	2.89 ± 1.01	0.51	−0.6	−1.31	2.51	0.46 ± 0.35	0.33 ± 0.15	0.24	−0.13	−0.1	0.36
	T2		6.24 ± 3.11	6.82 ± 3.06	0.65	0.58	−3.19	2.03	0.72 ± 0.30	0.53 ± 0.23	0.1	−0.19	−0.04	0.42
	T3		11.82 ± 3.53	10.49 ± 4.66	0.44	−1.33	−2.17	4.82	1.11 ± 0.26	0.81 ± 0.34	0.023*	−0.3	−0.05	0.56
Lower canines	T1	2.75 ± 1.76		3.61 ± 1.68	0.23	0.86	−2.32	0.6	0.23 ± 0.47	0.23 ± 0.20	0.99	0	−0.3	0.31
	T2		7.35 ± 1.63	7.62 ± 2.28	0.74	0.27	−1.95	1.41	0.62 ± 0.45	0.54 ± 0.39	0.64	−0.08	−0.28	0.44
	T3		9.90 ± 1.77	11.55 ± 3.31	0.15	1.65	−3.89	0.6	1.24 ± 0.60	0.98 ± 0.41	0.23	−0.26	−0.18	0.7

Regarding root resorption, both control and FLC groups showed a statistically significant decrease in canine root length. The resorption in the upper and lower control sides were 0.68 mm (p = 0.002) and 0.56 mm (p = 0.003), respectively, and in the upper and lower FLC sides were 0.59 mm (p = 0.001) and 0.63mm (p < 0.001), respectively. However, no significant differences were observed between FLC and control sides in the upper and lower canines at baseline and after 3 months of retraction in the measurement of root resorption (p > 0.05) (Table 5).

**Table 5:** Comparison between and within each group in terms of root resorption before and after canine retraction. Paired t-test within group and Student t-test between groups; T0 immediately before canine retraction; T3 after canine retraction CI: Confidence interval; LL: Lower limit; UL: Upper limit *Statistically significant at p ≤ 0.05

Canine Root length (mm)							
		CR Control Mean (mm) ± SD	FLC Mean (mm) ± SD	p value	Mean Diff (mm)	95% CI	
						LL	UL
Upper canines	T0	26.37 ± 0.87	26.25 ± 1.15	0.39	−0.12	−0.36	0.59
	T3	25.69 ± 1.02	25.66 ± 1.26	0.47	−0.03	−0.34	0.4
p value		0.002*	0.001*				
Mean Diff (mm)		−0.69	−0.54				
95% CI		(−1, −0.32)	(−0.79, −0.27)				
Lower canines	T0	25.34 ± 0.91	25.42 ± 1.07	0.43	−0.08	−0.33	0.18
	T3	24.78 ± 1.12	24.79 ± 1.09	0.49	−0.01	−0.42	0.39
p value		0.003*	<0.001*				
Mean Diff (mm)		−0.56	−0.63				
95% CI		(−0.88, −0.24)	(−0.86, −0.39)				

Periodontal assessment data revealed non-significant differences in probing depth within each group and between FLC and control upper and lower groups at baseline and after three months of retraction (p > 0.05) (Table 6). Additionally, there was a significant difference in the plaque index in the upper FLC group between baseline and after three months (p = 0.046). However, no significant difference was found in plaque index or gingival index in all other groups (p > 0.05) (Table 7). Regarding pulp vitality, all canines showed positive vitality response at baseline and after three months of the intervention. 

**Table 6:** Comparison between and within each group in terms of probing depth before and after canine retraction. Paired t-test for within-group comparison,  and student t-test for comparing between groups. T0: immediately before canine retraction; T3: three months after canine retraction SD: standard deviation; CR: conventional retraction; FLC: flapless laser corticotomy

Probing depth (mm)					
		CR Control Mean (mm) ± SD	FLC Mean (mm) ± SD	p-value	Mean Difference (mm)
Upper canines	T0	1.38 ± 0.48	1.67 ± 0.49	0.15	0.29
	T3	1.42 ± 0.47	1.79 ± 0.45	0.06	0.37
p-value		0.67	0.19		
Mean Difference (mm)		0.04	0.12		
Lower canines	T0	1.54 ± 0.45	1.67 ± 0.49	0.52	0.13
	T3	1.83 ± 0.58	1.71 ± 0.54	0.59	-0.12
p-value		0.07	0.72		
Mean Difference (mm)		0.29	0.04		

**Table 7:** Comparison within each group in terms of plaque index, gingival index before and after canine retraction Wilcoxon signed-rank test; T0: immediately before canine  retraction; T3: three months after canine retraction CR: conventional retraction; FLC: flapless laser corticotomy * Statistically significant at p ≤ 0.05

		T0 (Mean ± SD)	T3 (Mean ± SD)	p-value
Plaque index				
Upper canines	FLC	0.33 ± 0.49	0.67 ± 0.65	0.046*
	CR Control	0.42 ± 0.67	0.67 ± 0.89	0.083
Lower canines	FLC	1.08 ± 0.67	0.92 ± 0.79	0.414
	CR Control	0.92 ± 0.79	0.92 ± 0.90	1
Gingival index				
Upper canines	FLC	0.50 ± 0.67	1.08 ± 1.0	0.07
	CR Control	0.75 ± 0.75	1.08 ± 0.67	0.206
Lower canines	FLC	0.92 ± 0.90	0.92 ± 0.79	1
	CR Control	0.92 ± 0.79	1.25 ± 1.14	0.102

## Discussion

This study was designed to evaluate the effects of FLC on the rate of upper and lower canine retraction and other possible side effects such as canine rotation, molar anchorage loss, root resorption, periodontal effects, and pulp vitality. We conducted this study on 14 patients with a mean age of 20.4 ± 2.5 years. A split-mouth design was selected to eliminate a lot of the inter-subject variability because each patient acts as their own control. Compared with parallel studies in which patients receive only one intervention, this design helps in increasing the study power or decreasing the number of subjects required [37].

Moreover, our sample was selected according to our inclusion criteria, which tried to eliminate most of the cofounders that may have affected the results. Regarding the subjects’ age, patients ≥18 years were recruited as corticotomy procedures in adolescents might be rarely indicated; this is attributed to a relatively higher rate of tooth movement. Alikhani et al. discovered that adults showed a significantly higher level of osteoclast activity and cytokine release but had a significantly slower rate of orthodontic tooth movement [38]. Hence, it was obvious that older patients might benefit from the corticotomy procedure owing to the reduced resistance of hard tissues and increased orthodontic tooth movement.

This study recruited cases complaining of dental class I with bimaxillary dentoalveolar protrusion, requiring four premolar extraction. This selection was based on our objective to evaluate the rate of canine movement in upper and lower canines that had undergone FLC. Most studies conducted to assess canine retraction acceleration selected only the upper canines for study; hence, they enrolled patients with class II div 1 malocclusion [30,31,33]. The reason why most studies selected the maxillary arch may be that it is preferred for the analysis because of the presence of distinctive landmarks on the palate that allows for more straightforward measurement. We attempted to overcome this limitation by using the third rugae area as a stable structure for superimposition and by creating a common reference plane for both the upper and lower models [39].

Most studies evaluating canine retraction rates share some features with the methodology of this study. In this research, all cases were treated by one operator using fixed upper and lower pre-adjusted edgewise appliance (MBT) prescription with slot size 0.022" and a 0.017 x 0.025" SS archwire that served as a working wire due to its rigidity. This helped in controlling the tipping of the canines during retraction. Some studies used 0.016 x 0.022" SS to reduce friction and facilitate the movement of the canine but at the expense of more tipping [32]. Some researchers employed a 0.019 x0.025" SS archwire, which is undoubtedly a better option for controlling the tipping [31,33]. However, it allows less play between the bracket and the wire, which leads to higher friction and may negatively affect the rate of tooth movement. The extraction was performed after leveling and alignment and three months before canine retraction to avoid overlap between the possible acceleration of tooth movement from the FLC and the acceleration after a recent extraction that occurs due to high cellular activity [40].

In this study, sliding mechanics were selected to achieve more control of force level during the study, similar to many studies with the same interest as ours. However, the difference can be attributed to the force delivery system. We used a NiTi coil spring adjusted at 150 gm as many studies proved that it was more effective in obtaining space closure [41,42]. This effectiveness made the coil spring the preferred method for force delivery in many studies investigating the rate of canine retraction. [1,31,32,43,44]. However, other researchers preferred elastic chains for canine retraction [30,33], which might be less expensive and easier to use but suffer from rapid force decay, requiring frequent adjustments to sustain the force levels.

We used mini-screws to augment the posterior anchorage, as it is considered the preferred method for anchorage reinforcement in maximum anchorage cases [45]. We used the indirect method of anchorage to allow better sliding mechanics. Both indirect [44,33] and direct [43,46] methods of mini-screw anchorage have been used in past research to assess the canine retraction acceleration rate. Evidence highlights that loaded mini-screws can provide a successful anchorage both directly and indirectly. Moreover, indirect mini-screw anchorage enabled better sliding mechanics than direct anchorage [47].

In this study, we used an Er-Cr: YSGG laser device with a wavelength of 2780 nm. The average power for bone cutting was 4.5 W at a pulse repetition rate of 40 Hz, with the energy per pulse being 112.5 MJ. Our parameters differed from those used by Seifi et al. [29], who used the same laser on eight New Zealand male rabbits to deliver energy of 300 MJ at pulse rates of 20 Hz. Moreover, Moahmoudzadeh et al. utilized the same laser for bone cutting with parameters of 3.5 W power and 30 Hz frequency [32]. Other researchers have employed different types of lasers to perform the corticotomy. Erbium, Yttrium crystal, Aluminum, and Garnet-enriched (Er: YAG) lasers were used by Salman et al., who did not report their laser parameters [30]. Another study by Alfawal et al. set up the parameters for hard tissue as 200 MJ, 12 Hz, and 3 W [31]. Moreover, Jaber et al. set parameters for hard tissue as 200 MJ and 15 Hz [33]. It seems that a wide variety of parameters have been used, starting from 200 mJ to 300 mJ, and a pulse rate from 12 Hz up to 30 Hz with no specific recommendations.

Regarding surgical technique, we performed FLC as a buccal series of six holes, with three mesial holes and three distal holes parallel to the long axis of the canine that were 2-3 mm apart and had a depth of 3 mm into the cancellous bone. Our technique was similar to the technique performed by Salman et al., who performed buccal series of four holes that were both mesial and distal to canine and 2-3 mm apart, with 1.5 mm diameter and 3 mm depth [30]. Moahmoudzadeh et al. modified this technique by making buccal vertical cuts on mesial and distal root surfaces parallel to the canine’s long axis 1 mm below the alveolar crest and extending up to the mucogingival junction, with a depth of 2-3 mm into the cancellous bone [32]. Alfawal et al. used another technique in which they performed five buccal perforations between the canine and second premolar that were 1.5-2 mm apart, 1.3 mm wide, and 3 mm in depth [31]. Jaber et al. made the highest number of holes as they performed eight buccal perforations in attached mucosa and drilled four distal holes and four around the canine that had a width of 1 mm and were 3 mm deep [33].

In this study, we started canine retraction immediately after the surgical procedure in agreement with many studies that recommended that orthodontic force not be delayed by more than one to two weeks post surgery to guarantee utilization of the RAP [10,12,17]. The upper and lower canines in the experimental group showed a slightly higher but non-significant rate of tooth movement than those in the control group in the first two months of retraction, with a mean difference of approximately 0.11 mm and 0.21 mm (p = 0.18, p = 0.36) in the first and second months, respectively between the experimental and control groups. This slight increase in the rate of tooth movement in the first two months was in agreement with many studies that suggested that RAP begins within a few days of operation, typically peaks at one to two months, and lasts for the first three to four months post surgery after which the speed of tooth movement declines to the pre-surgery level [14,17,48].

Moreover, the analysis showed a statistically non-significant difference between all groups (FLC and control) in the change and monthly rate of canine retraction at all time points. This indicated that FLC could not facilitate a significant increase in the rate of canine movement. These results were very similar to those reported by different split-mouth RCTs. For example, Alkebsi et al. [46] and Aboalnaga et al. [44] who studied the effect of micro-osteoperforations and Mistry et al. [49] who studied the effect of low-level laser therapy found non-significant differences between their experimental and control groups.

However, our results were contradictory to other studies that reported a significant increase in the rate of canine movement with FLC [30-33]. The current results that highlighted that FLC could not accelerate the rate of tooth movement can be explained by considering that the FLC technique is a relatively minimally invasive procedure. A total of six holes, three distal and three mesial, were drilled into the canines in this study, which might be an inadequate osseous insult that is unable to fully trigger the RAP, especially if we add the results of Yang et al., who reported that mesial cuts in the labial surface exert a minor influence on dentoalveolar structures, while distal corticotomy cuts closer to the canine root and may be more useful in corticotomy-facilitated canine retraction [50]. Murphy et al. revealed the criticality of boney insults to activate cellular reactions that lead to tooth movement acceleration [51]. This could justify why the current results were similar to the results of the RCTs that performed three distal MOPs and discovered that their procedures were insufficient to accelerate the rate of canine movement [44,46]. Moreover, the inadequate boney insults also justify the contradictory results with the other studies that had more invasive procedures, such as Salman et al. [30] who drilled eight holes (four distal and four mesial), Alfawal et al. [31] who drilled five distal holes, Moahmoudzadeh et al. [32] who performed mesial and distal linear cuts), and Jaber et al. [33] who drilled right holes (four distal and four around the canine). It is worth mentioning that the previously mentioned studies had adopted different methods for assessing canine retraction. Salman et al. [30] did not report the exact methods or landmarks used for assessment, Alfawal et al. [31] used calibrated photos to assess canine retraction distance, and Jaber et al. [33] used direct intraoral measurements. This difference in assessment methods might have influenced the results. Moreover, the lack of data regarding the protocol for FLC and laser parameters might have influenced the outcome. It was reported that acceleration of orthodontic tooth movement depends on the protocol as the low dosage of low-level laser therapy increases the amount of tooth movement, while a higher dosage results in an inhibitory effect [52].

Secondary outcomes

Regarding canine rotation, the results revealed a statistically non-significant difference between all groups (FLC and control) in canine rotation change at all time points. The same results were obtained by Alkebsi et al. who found non-significant differences between the experimental and control groups regarding canine rotation, as they used steel ligature over rigid SS wires for canine retraction, which was considered a limiting factor for canine rotation during retraction [46]. Moreover, Alfawal et al. also found an insignificant increase in canine rotation between the experimental and control groups; however, their results showed higher amounts of total rotation during the three months of retraction [31]. They reported approximately 17.7 degrees in the experimental group and 16.45 degrees in the control group, as they used an elastic module (O-tie) for canine ligation. This justifies why they showed a higher amount of rotation within the groups.

Regarding anchorage loss, our results revealed statistically insignificant differences between the control and FLC groups. This was also observed in similar studies [31,32]. The total amounts of molar anchorage loss in maxillary arches in the current study were 0.81 ± 0.34 and 1.11 ± 0.26 mm for the experimental and control groups, respectively. The same for mandibular arches were 0.98 ± 0.41 and 1.24 ± 0.60 mm for the experimental and control groups, respectively. Our results were in agreement with Zhang et al. who reported a maxillary molar mesial movement of about 0.83 mm with indirect mini-screw anchorage [47]. A Cochrane review conducted by Skeggs et al. to compare the reinforced implant versus traditional means of anchorage found significant differences in anchorage loss between both groups, and the mean change in the implant anchorage group was 1.5 mm [53]. In our study, the highest amount of anchorage loss was observed in the lower control group, which was approximately 1.24 ± 0.60 mm; however, these results were considered clinically insignificant (<1.5 mm) and still lie within the values of the maximum anchorage definition. Anchorage loss in our study was less than that in the results of Alfawal et al., who reported anchorage loss of approximately 1.6 mm in the FLC group and 1.88 mm in the control group as they used transpalatal arch (TPA) for anchorage [31].

Regarding root resorption, a statistically non-significant difference was found in canine root length between the groups after treatment. These results agreed with those of previous canine acceleration studies that found a non-significant difference between the experimental and control groups [44,46]. These results eliminate the possibility that FLC might cause higher root resorption.

Interestingly, our results indicated a statistically significant decrease in canine root length after treatment (p < 0.001*) in all groups (upper and lower FLC and control). For maxillary canines, the average amount of root resorption was 0.54 and 0.69 mm for experimental and control groups, respectively, and the same for mandibular canines was 0.63 and 0.56 mm for experimental and control groups, respectively. None of the studies that evaluated FLC investigated root resorption; however, other studies that investigated corticotomy such as Kabbur et al. [54] and MOPs such as Aboalnaga et al. [44] reported non-significant differences between root length before and after treatment within the MOP and control groups. On the other hand, our results agreed with Alkebsi et al. who found significant root resorption in both the MOP and control groups [46]. Even though the statistical analysis of our results highlighted a statistically significant change in root length, this change was less than 1 mm (0.56-0.68 mm), which can be considered clinically insignificant.

Periodontal assessment in the current study revealed a non-significant difference in probing depth (mm), the plaque score (except in the upper experimental), and the gingival score after treatment in all groups. This was in agreement with most of the studies that investigated corticotomies and MOPs in canine acceleration [30,43,46].

The vitality of all canines was examined before and immediately after canine retraction using a cold thermal test. All canines showed a positive response to the cold thermal test before and after canine retraction. Our results were consistent with most previous research [27,55,56]. 

In the days immediately following an accelerated orthodontic intervention, patients undergoing surgically-assisted orthodontic acceleration may experience pain or discomfort [18]. Future studies should take into account the patient-reported outcomes following acceleration, such as pain, discomfort, and swelling, which were not evaluated in the current trial.

Limitations

Although the design of this study tried to eliminate all confounders that may affect the results, it still had the following limitations: (1) most of our sample was composed of female subjects, which made it difficult to detect the different responses (if found) between males and females, (2) The measurement of root resorption was conducted using CBCT before the start of treatment and after retraction. It would have been better if CBCT had been taken immediately before and after canine retraction. However, this was not possible to avoid overexposing the patient to a considerably high radiation dose of X-ray, (3) Inflammatory markers were not assessed during different time points, and (4) Finally, the digital casts at different time points were made by scanning plaster models and not by direct scanning via intraoral scanners.

Generalizability 

The FLC technique was safe and minimally invasive; however, the results revealed the ineffectiveness of this method for tooth movement acceleration. Further investigations are required to evaluate different laser parameters, the number and position of decortication with larger sample sizes, and multi-center participation to validate these results and increase their generalizability. 

## Conclusions

The FLC procedure performed in this study was unable to accelerate the rate of upper and lower canine retraction. No statistically significant differences were observed between the FLC and control groups regarding canine rotation and the first molar anchorage loss at the end of treatment. No statistically significant differences were found between the FLC and control groups regarding root resorption, and clinically insignificant amounts of resorption were found within each group. Finally, the FLC has no adverse effects on periodontal health and pulp vitality.
